# Leveraging graph neural networks for supporting automatic triage of patients

**DOI:** 10.1038/s41598-024-63376-2

**Published:** 2024-05-31

**Authors:** Annamaria Defilippo, Pierangelo Veltri, Pietro Lió, Pietro Hiram Guzzi

**Affiliations:** 1 Dept. Medical and Surgical Sciences, Magna Graecia University of Catanzaro, Catanzaro, Italy; 2 DIMES Department of Informatics, Modeling, Electronics and Systems, UNICAL, Rende, Cosenza, Italy; 3 Department of Computer Science and Technology, Cambridge University, Cambridge, UK; 4 Dept. Medical and Surgical Sciences, Magna Graecia University of Catanzaro, Catanzaro, Italy

**Keywords:** Component, Formatting, Style, Styling, Insert, Computational science, Health policy

## Abstract

Patient triage is crucial in emergency departments, ensuring timely and appropriate care based on correctly evaluating the emergency grade of patient conditions. Triage methods are generally performed by human operator based on her own experience and information that are gathered from the patient management process. Thus, it is a process that can generate errors in emergency-level associations. Recently, Traditional triage methods heavily rely on human decisions, which can be subjective and prone to errors. A growing interest has recently been focused on leveraging artificial intelligence (AI) to develop algorithms to maximize information gathering and minimize errors in patient triage processing. We define and implement an AI-based module to manage patients’ emergency code assignments in emergency departments. It uses historical data from the emergency department to train the medical decision-making process. Data containing relevant patient information, such as vital signs, symptoms, and medical history, accurately classify patients into triage categories. Experimental results demonstrate that the proposed algorithm achieved high accuracy outperforming traditional triage methods. By using the proposed method, we claim that healthcare professionals can predict severity index to guide patient management processing and resource allocation.

## Introduction

Emergency department (ED) management faces a significant challenge in handling the influx of patients. Properly managing queues can help enhance hospital quality, contain costs, and ensure proper reimbursement^1^. The management of patient queues in the ED adheres to rules defined by national healthcare system laws. In Italy, healthcare services are accessible to all citizens without restrictions. However, in recent years, demographic shifts and pandemics have led to frequent overcrowding in emergency departments, underscoring the relevance of this issue^2–5^. Managing queues in EDs is critical, considering both economic and social aspects, as unfair patient prioritization can lead to potentially fatal outcomes. Prioritization in patient management, regarding treatment and evaluation, is facilitated through a process called triage. Triage involves gathering information during an initial rapid patient assessment, measuring vital signs, and assigning an emergency level to each patient to determine treatment priority. Each priority level corresponds to an emergency code, guiding treatment protocols.

**Figure 1:**
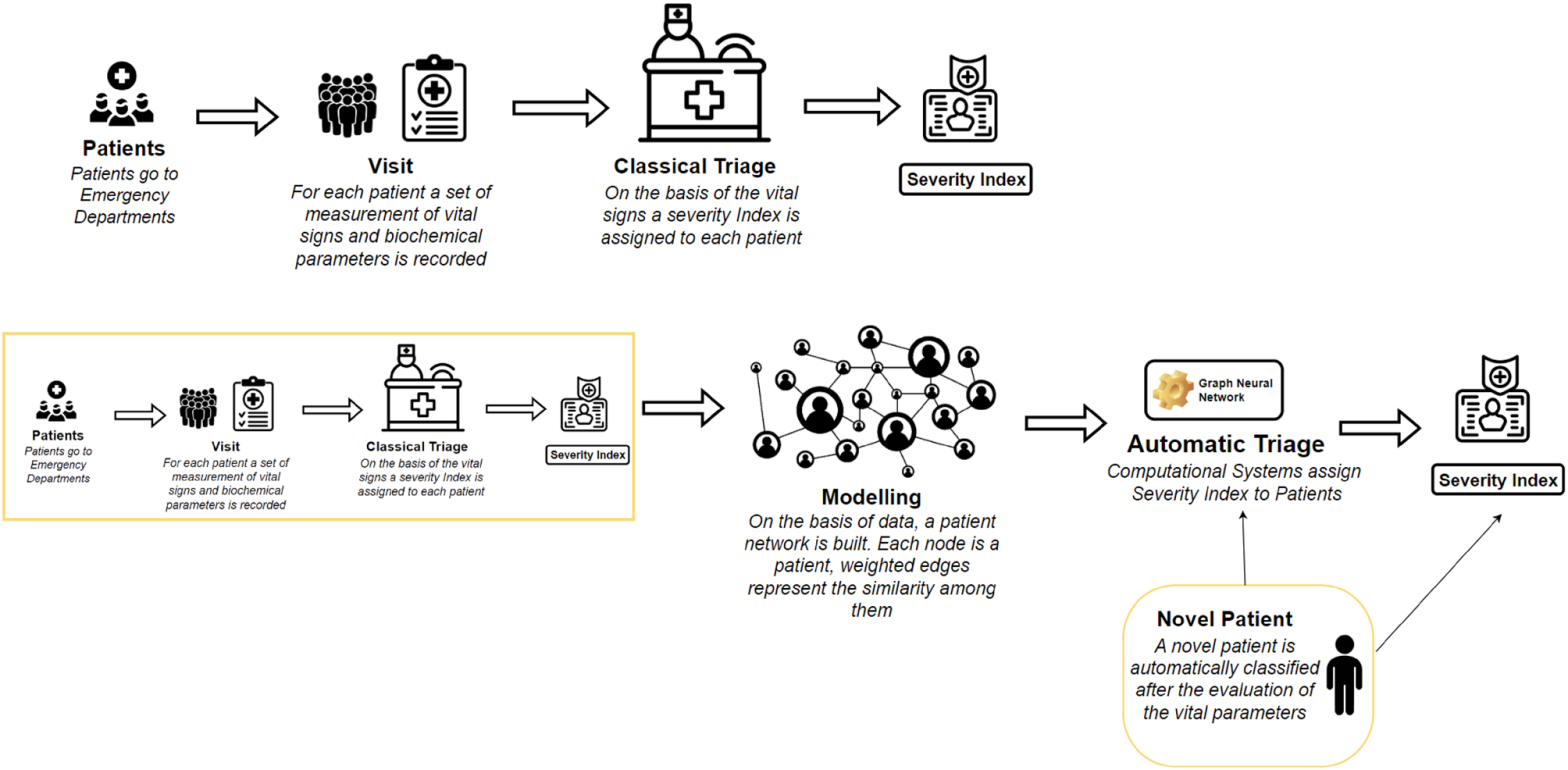
Figure compares automatic vs traditional triage systems. In the traditional triage system, patients go to the Emergency Departments for admission. Vital signs and biochemical parameters are evaluated, and an emergency code is assigned. In the scenario, we envision data from previous admissions being used initially to build a network representing patient similarity. The network is then used to learn a Graph Neural Network to classify patients in a latent space (the assignment of the severity index is translated into a multiclass classification problem). Finally, each patient entering the emergency room is automatically classified after acquiring clinical data.

Therefore, proper queue prioritization for new patients (i.e., classification) depends on patients’ clinical and health conditions to ensure appropriate treatment prioritization for those arriving at the emergency department^6,7^.

Various methods and techniques for defining triage have been established and adopted on a large scale^8,9^. In Italy, the rules for the triage process are outlined in the Italian healthcare management guidelines, which define triage in four phases:

1. Immediate Evaluation Phase (known as “on the door”): Identifying patients’ conditions to screen those needing immediate intervention.
2. Subjective and Objective Evaluation Phase: Assessing conditions through interviews and clinical analysis to detect signs and vital parameters.
3. Triage Decision Phase: Assigning a priority code to each patient and organizing waiting queues.
4. Re-evaluation Phase: Periodically analyzing patients to confirm or modify priority codes.

Considering the global scenario, several widely adopted triage systems include the Canadian Triage and Acuity Scale (CTAS), the Australasian Triage System (ATS), the Manchester Triage System (MTS), the Emergency Severity Index (ESI), the Korean Triage and Acuity Scale (KTAS), the Taiwan Triage Acuity Scale (TTAS), and the South African Acuity Scale (SAAS)^10–17^. These systems typically involve the categorization of patients based on their conditions and directing them to different areas of the ED.

The application of triage rules relies heavily on operator decisions (nurses and physicians) interacting with patient parameters and data. Triage assessment considers various factors determining the urgency of each incoming patient. However, manual triage approaches share common limitations, including inconsistencies in assessment due to various factors influencing patient urgency^18^. Consequently, there’s been a push towards defining and implementing computational-based triage code assignment mechanisms to minimize human-related errors^19,20^.

Many authors have explored the potential of automated systems based on computational intelligence to improve triage by suggesting emergency codes^19,20^. Furthermore, machine learning (ML) and artificial intelligence (AI) algorithms have demonstrated the ability to analyze electronic medical records (EMRs) and unstructured patient data from sensors or wearable devices^21,22^. As triage systems rely on patient data observation, ML and AI have been investigated to enhance classical clinical scoring systems^9^.

Numerous prediction models have been developed to refine the triage process, offering more nuanced patient stratification within traditional groups and improving clinical outcomes^8,9^. For instance, Olivia et al.^23^ employed supervised learning algorithms to predict patients’ medical conditions accurately. Similarly, Caicedo-Torres et al.^24^ used machine learning techniques in a pediatric ED to determine patients requiring rapid admission and pediatric treatment. Joseph et al.^25^ conducted a comprehensive study using deep learning to identify critically ill patients based on limited triage information. These studies underscore the significant potential of machine learning in managing patient triage in the emergency department.

Existing prediction models use data collected at triage, including demographic information, vital signs, primary complaints, nursing observations, and initial diagnostics^8,9^. Some models also incorporate historical data such as patient access frequencies and medical records. Thus, leveraging patient data from ED admission and treatment could enrich and improve triage and patient management processes.

Often, data obtained later in a patient’s ED visit, such as laboratory tests and diagnoses, are more effective in predicting admissions. Although we do not have access to the complete patient medical record containing all the features mentioned, in our study we utilised variables directly obtainable during the triage phase (such as blood pressure) and other patient history information (such as diabetes pedigree). It is necessary to point out that these features have been associated as attributes of the graph nodes to take them into account in the embedding and prediction phase improving the results. Our proposal could be adapted in case of any other available medical features to be included for the node classification.

Identifying similarities among patients’ conditions and their previous visits could aid in predicting admissions at the triage stage. Current solutions often employ broad categories for chronic conditions, but enriching electronic health record (EHR) data to train models could enhance prediction accuracy^26–28^. Although approaches using gradient boosting and deep neural networks improve prediction efficacy, explicit modeling of patient similarity is lacking in existing methods^29^. In fact, most of the existing approaches in the literature treat data in tabular format or model patient features in graph form, but possible similarities among patients are not taken into account. Instead, consider the similarity between patients could influence the model’s performances and enhance the model’s ability to capture hidden patterns^30^. Modelling data as graphs and using embedding has been shown good performances in node classification task. Graphs contains properties of the nodes (also referred to as attributes) and structure information (e.g. similarity among nodes) which help in classification^31,32^.

We propose to model patients as graphs and then to use graph embedding and classification techniques to develop a novel clinical algorithm based on artificial intelligence and network science for assigning patient priority. Clinical patient data, including analytical and subjective observations, are extracted from patient records and undergo preprocessing to identify noise and outliers. Each patient is represented as a graph node, with edges indicating similarity among observation data. The graph is then embedded into a latent space, and patients are classified into risk groups using a node classification algorithm, as depicted in Fig. 1.

Given the potential of machine learning in medicine, and in particular in managing patient triage in the emergency department, we presented the possibilities to model patients in different similarity networks explored with three Graph Neural Network architectures. Existing prediction models utilize data collected at triage in the form of tabular data. However, identifying similarities among patients could aid in predicting admissions at the triage stage^30^. Therefore, we propose modelling not only the patient’s features in the networks, but also the patient entity with its related attributes and weighted edges showing the similarities between patients.

In a classical triage system, patients requiring admission to ED are classified into severity levels using standard algorithms that produce a severity index based on the patient data. Such data may be obtained by the nurse’s observation of the patient and evaluation of biochemical parameters. Such data are usually stored in hospital registries and EHRs. We start by considering these data to build a patient similarity network where each patient is a node. The edges among patients are weighted by calculating the similarity of patients based on their data. Each node is also labelled with the previously assigned severity level. The network is embedded into a latent space, and a node classifier is trained. Once that the classifier is trained, each new patient can be automatically assigned to a severity index by using the classifier as summarised in Fig. 1.

To sum up, our solution is based on modeling patient similarity using networks (each patient is a node of the network) for the application of Graph Neural Networks (GNNs) for node classification. GNNs allow the possibility to take advantage of tabular data modeled in networks in order to discover hidden relationships in the data. GNNs not only take into account the variables present in the dataset modeled as node attributes, but they exploit the topology of the graph by considering the links between patients (i.e. between nodes). Despite data on a single patient may be scarce, similarity with other patients could aid in correct prioritisation. In addition, our approach not only provides a novel design but also offers reusable and available code, whose accessibility is specified in the dedicated section.

Our approach offers an inductive embedding, enabling the addition of novel patients to the graph for classification^33,34^. We evaluated our pipeline using public data to demonstrate its effectiveness and improvement over state-of-the-art approaches.

## Related work

Triage is a critical and systematic emergency department (ED) process. It helps prioritize patients based on the severity of their condition^9^, the urgency of their need for care, and the availability of resources within the healthcare facility. The goal is to ensure that patients who need immediate attention receive it promptly^16^ while those with less critical needs are attended to in an order that maximizes the overall efficiency and effectiveness of emergency medical services.

The triage process in an emergency department involves several key steps and principles^35^, which are as follows. Upon arrival at the ED, each patient undergoes an initial assessment by a triage nurse or a trained healthcare professional to assign a urgency categorization, ranging from immediate life-saving intervention needed (highest priority) to non-urgent care (lowest priority)^13^^14^. This assessment is designed to quickly gather critical information about the patient’s condition, including the chief complaint, vital signs (such as temperature, blood pressure, heart rate, and respiratory rate), and a brief history of the present illness or injury.

Triage categorization directly influences the allocation of resources^14^, because triage is not a one-time assessment but a continuous process. Patients with the highest urgency levels are treated immediately and often directed to specialized areas within the ED equipped to handle severe cases (e.g., resuscitation rooms). Those with lower urgency levels may wait longer and be seen in order of priority based on their triage category^16^. It is not excluded that patients’ conditions can change, necessitating a reassessment and potential re-categorization of their urgency level^15^.

Triage involves ethical considerations, such as fairness, equity, and the principle of doing the most good for the greatest number of people^36^. Healthcare professionals must make unbiased decisions based on clinical urgency and the potential for benefit from medical intervention rather than factors like financial status, age, or social position^35^. The triage process faces various challenges, including overcrowding in emergency departments, fluctuating patient volumes, and limited resources. Effective triage requires flexibility and the ability to adapt to changing circumstances, such as public health emergencies or disasters, which may necessitate modifications to triage protocols and prioritization strategies^36^. Despite their worldwide adoption and diffusion, triage systems are affected by some common problems^35^, such as dependence on subjective medical staff assessment and the possibility of having many missing variables.

Conversely, in an automatic triage system, the categorization of patients is fully automated and the severity level is determined by computer algorithms as summarised in^26^. Automatic (or computing-based), triage systems present some advantages such as: (i) stability of assignment^26^, (ii) filtering out noise in the patient variables, (iii) modelling and analyse patient similarity, (i.e. by modelling the set of patients in a network which evidences patient similarity^26,37–40^; (iv) avoiding patients under triaged into low severity levels^41^; (vi) avoid of racial, gender, age bias^42^. Literature contains many approaches of the use of machine learning for patients emergency classification, as reported in the Tables 1 and 2.

Singh et al.,^43^ developed a cascading classifier for psychiatric patient triage, achieving high accuracy and reducing expert effort. Graca (2023) highlighted the potential of machine learning in ICU triage and patient transfer during crises, such as the Covid-19 pandemic. Olivia et al.,^23^ and Yan et al.,^44^ both emphasized the significance of machine learning in emergency department triage, focusing the effectiveness of Support Vector Machine, Naive Bayes and Decision Tree models.

It should be noted that the examples just mentioned are only some of the methods present in the literature. Other examples of significant interest are shown in Table 1, showing aims and methods to provide an overview of existing solutions. In addition, further details on the topic are available in^8^.

The methods presented are based on ML (or DL) algorithms with similar aims and tasks of our solution. However, as mentioned in the introduction section, patient data are conventionally managed in tabular formats, differently from our approach based on similarity networks for representing patient data. Although our solution is innovative, it is appropriate to provide an overview of some solutions that exploit network modelling for the analysis of ED patient data. Many deep learning-based medical prediction methods are focused on the patient’s individual information. Due to missing data, noise, and incompleteness a single EHR cannot provide complete health information^45^. In this regard, considering patients similarities could enhance models’ performances.

Table 2 summarizes some network-based approaches for the managing of ED by outlining, not only the purpose and the methods, but also the key features that differentiate them from our proposal. For example, although it is not a paper strictly related to ED, Liu et al.,^46^ address the possible relationships between symptoms and diagnoses in the medical field modelling chatbot data in multi-relation graph with multiple type of nodes and edges. It shows how graph-based methods are flexible, capturing intricate health-related data structures effectively. Ying et al.,^45^ propose MERGE, a Multi-graph attentive Representation learning framework for the integration of group information from similar patients for medical prediction. Valls et al.,^47^ analyse the use of GNNs on Knowledge Graphs, highlighting the importance of domain knowledge in GNN connectivity for link predictions in the clinical triage context. Still in the background of application in emergency departments, Tong et al.,^30^ present the modelling of patients in similarity networks with costumed pairwise similarity score, using LSTM-GNN for the prediction of mortality and length of stay.

**Table 1:** Exploring some machine learning approaches for analyzing patients in emergency department^1^.

Title	Aim	Methods
Machine learning based electronic triage for emergency department^23^	Predicting the patient’s medical condition, given their signs and symptoms	NB, SVM, DT and, NN classification models
A machine learning model for triage in lean pediatric emergency departments^24^	Pediatric ED to correctly predict which patients should be ad-mitted, given their signs and symptoms	LR, SVM with Polynomial and Gaussian kernels and the MLP NN
Deep-learning approaches to identify critically Ill patients at emergency department triage using limited information^25^	Predicting critical illness at triage	Four progressively complex deep-learning models
Machine-Learning-Based Electronic Triage More Accurately Differentiates Patients With Respect to Clinical Outcomes Compared With the Emergency Severity Index^26^	Predicting likelihood of acute outcomes enabling improved patient differentiation	E-triage composed of a random forest model
A Novel Interpretable Deep-Learning-Based System for Triage Prediction in the Emergency Department: A Prospective Study^27^	Predicting hospitalization based on prospectively collected data in the ED, including vital signs and chief complaints	Interpretable novel triage prediction system
Predicting hospital admission at emergency department triage using machine learning^28^	Predicting hospital admission at the time of ED triage using patient history in addition to information collected at triage	Nine binary classifiers using LR, XGBoost, and DNN
Machine Learning and Initial Nursing Assessment-Based Triage System for Emergency Department^37^	Predicting adverse clinical outcome in ED based on initial nursing assessment (INA)	Four classifiers using LR and a DL model
Machine Learning-Based Prediction of Korean Triage and Acuity Scale Level in Emergency Department Patients^38^	Predicting the KTAS level	Logistic regression, random forest, and XGBoost
Machine learning models predicting undertriage in telephone triage^41^	Predicting undertriage in the prehospital setting and identifying the predictors of risk factors associated with undertriage	SVM, Lasso Regression, RF, XGB, and DNN
A Racially Unbiased, Machine Learning Approach to Prediction of Mortality: Algorithm Development Study^42^	Minimizing bias in in-hospital mortality predictions between white and nonwhite patient groups	XGBoost, a gradient boosting technique
Machine learning for psychiatric patient triaging: an investigation of cascading classifiers^43^	Triaging psychiatric patients using textual patient records	One-class-at-a-time approach, a multistage cascading classifier for psychiatric patient triage
Technology Road Mapping of Two Machine Learning Methods for Triaging Emergency Department Patients in Australia^44^	Exploring the application of ML to improve the triage process for ED patients in Australia	NB and NN on EHRs

^1^ NV = Naive Bayes, SVM = Support Vector Machine, DT = Decision Tree, NN = Neural Neteorks, LR = Logistic Regression, MLP = Multilayer Perceptron, XGBoost = Gradient Boosting, XGB = Gradient-Boosted Decision Tree, DNN = Deep Neural Networks, DL = Deep Learning, RF = Random Forest.

**Table 2:** Exploring some approaches for analyzing patients in Emergency Department using Networks.^2^.

Title	Aim	Methods	Main difference
Graph Network Techniques to Model and Analyze Emergency Department Patient Flow^48^	Modeling, storing, and analyzing patient journeys through the emergency department	Neo4j as a time-varying graph to model patient flows during ED stays	Graph network to model the sequence of events of a patient
Medical Triage Chatbot Diagnosis Improvement via Multi-relational Hyperbolic Graph Neural Network^46^	Analyzing the relationship between symptoms and potential diagnoses in healthcare datasets	Multi-relational Hyperbolic Diagnosis Predictor^3^ to build a disease predictive model	Multi-relational graph based on the chatbot data with three types of nodes along with two types of relations
Information Flow in Graph Neural Networks: A Clinical Triage Use Case^47^	Understanding how GNN parameters and domain knowledge influence the accuracy of predicting the appropriate patient cares	GNNs on KG to optimize link prediction for clinical triage	Link prediction as task, multi-relational KG as modelling method
Design of Intelligent Question Answering System for Hospital Online Triage based on Knowledge Graph^49^	Implementation of a medical question answering system to efficiently access relevant medical information by asking questions	Leverages KG in Ne04j to enhance the intelligence in the medical field	KG to construct a medical knowledge map for enhancing medical question answering systems
Predicting Patient Outcomes with Graph Representation Learning^30^	In-hospital mortality and length of stay prediction (patient outcome prediction tasks)	LSTM-GNN for: a hybrid model combining LSTMs for extracting temporal features and GNNs for extracting the patient neighbourhood information	Incorporating temporal features for outcome prediction, using costumed pairwise similarity score
MERGE: A Multi-graph Attentive Representation learning framework integrating Group information from similar patients^45^	Evaluating patient similarity and obtaining similar patient groups for clinical prediction tasks using EHRs	MERGE integrates group information from similar patients for clinical prediction tasks based on temporal EHRs	General medical prediction using dynamic and static patient feature to construct similar patient affinity graphs

^2^ KN = Knowledge Graph, GNN = Graph Neural Network, Neo4j = a graphing database, LSTM = Long Short-Term Memory network, EHR = Electronic Health Record

^3^ a novel multi-relational hyperbolic GNN based approach.

## Architecture of the proposed system

Our methodology leverages Graph Representation Learning applied to networks through Graph Neural Networks. Graph Representation Learning (GRL) is a method for encoding structural information of a graph into low-dimensional vectors. The mapping derived from projecting nodes or subgraphs of a graph into a vector space reflects the original graph’s structure. GRL enables the use of learned embeddings as feature inputs for downstream machine learning tasks, leveraging graph structures for various applications such as node classification, link prediction, and graph visualization. Many GRL approaches have been developed over time, but we have chosen Graph Neural Networks that deal with node labels based on inductive strategies.

**Figure 2:**
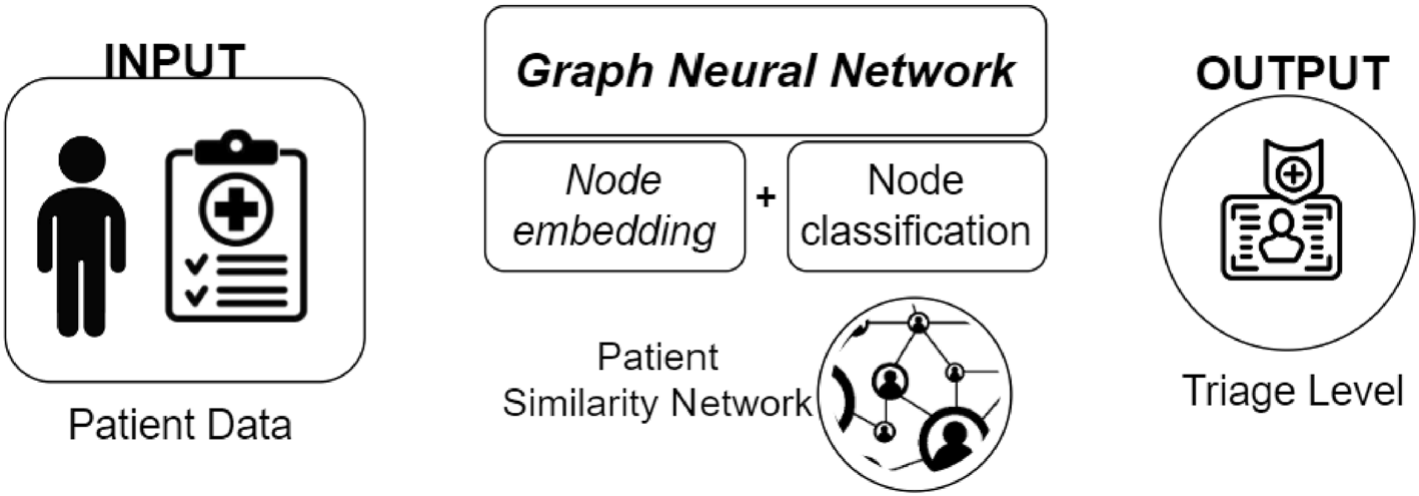
The architecture of the system.

Figure 2 describes the system’s architecture. The system receives patient data as input to build the patient similarity network, where each node is a patient, and the weighted edges model the similarity among them. The current implementation of the system uses four known measures for evaluating similarity: cosine similarity, Euclidean, Manhattan, and Minkowsky distances.

The network embedding (or graph representation learning) module is responsible for projecting the network into a latent space using node embedding. GRL projects each node into a separate point in a subspace while preserving the initial distance between nodes.

The node classification module is responsible for assigning a triage level to each new patient requiring the assignment. Both the graph representation learning module and node classification module leverage the computational intelligence of Graph Neural Networks methods. From the existing techniques for GRL, we select those that can deal with node labels based on inductive strategies. For instance, Graph Neural Networks are used for node embedding^50^ because they present two main advantages: (i) they take into account data related to nodes, (i.e. node features); (ii) they are inherently inductive, so they do not need to recalculate the whole embedding in case of any graph modification (e.g. node/edge insertion or removal)^34,51,52^. The current implementation uses three state-of-the-art methods:Graph Convolutional Networks^53^, GATv2Conv^54^, and GraphSage^55,56^, which in general have better performances compared to the state of the art.

Graph Convolutional Networks (GCNs)^57^ are specialized neural network architectures for processing data structured as graphs. These networks are particularly effective in scenarios where data points are interconnected, such as social networks, molecular structures, or communication networks. Unlike traditional convolutional neural networks that operate on grid-like data (e.g., images), GCNs leverage the graph structure to process data on nodes and their connections^57^. They apply convolution operations directly on the graph, aggregating information from a node’s neighbours, capturing the features of individual nodes and the complex relationships between them. The flexibility of GCNs in handling irregular graph structures makes them highly suitable for tasks like node classification, link prediction, and graph classification.

Graph Attention Networks (GATs)^58^ use an attention mechanism to dynamically determine the relevance of each neighbour’s features, allowing for a more nuanced and context-aware aggregation of neighbour information. This approach differs from traditional graph convolutional networks that aggregate neighbouring node features uniformly or predetermined.

In GATs, the attention mechanism assigns different weights to more relevant nodes in a neighbourhood, which is particularly useful in dealing with complex graph structures where the relevance of neighbouring nodes can vary significantly. This approach leads to more effective feature representation and learning. The implementation of GAT is defined as follows with two GATv2Conv layers:

- first layer with input dimension equal to 16 as the number of features, output dimension equal to 8 and 4 attention heads to implement the mechanism of attention.
- second layer with input dimension equal to the output dimension of the previous layer (hidden dimension * number of attention heads) to obtain the output of dimension 4 as the size of the target class.

GraphSAGE^55^ is a framework for generating node embeddings for large graphs. Unlike conventional graph-based methods, it samples and aggregates from a node’s local neighbourhood to efficiently scale to large graphs. It can perform aggregation using various functions and capture diverse neighbourhood structures effectively. GraphSAGE has inductive learning capability and is effective in classification, prediction, and recommendation systems for large-scale and dynamic graph data.

## Experimental results

**Figure 3:**
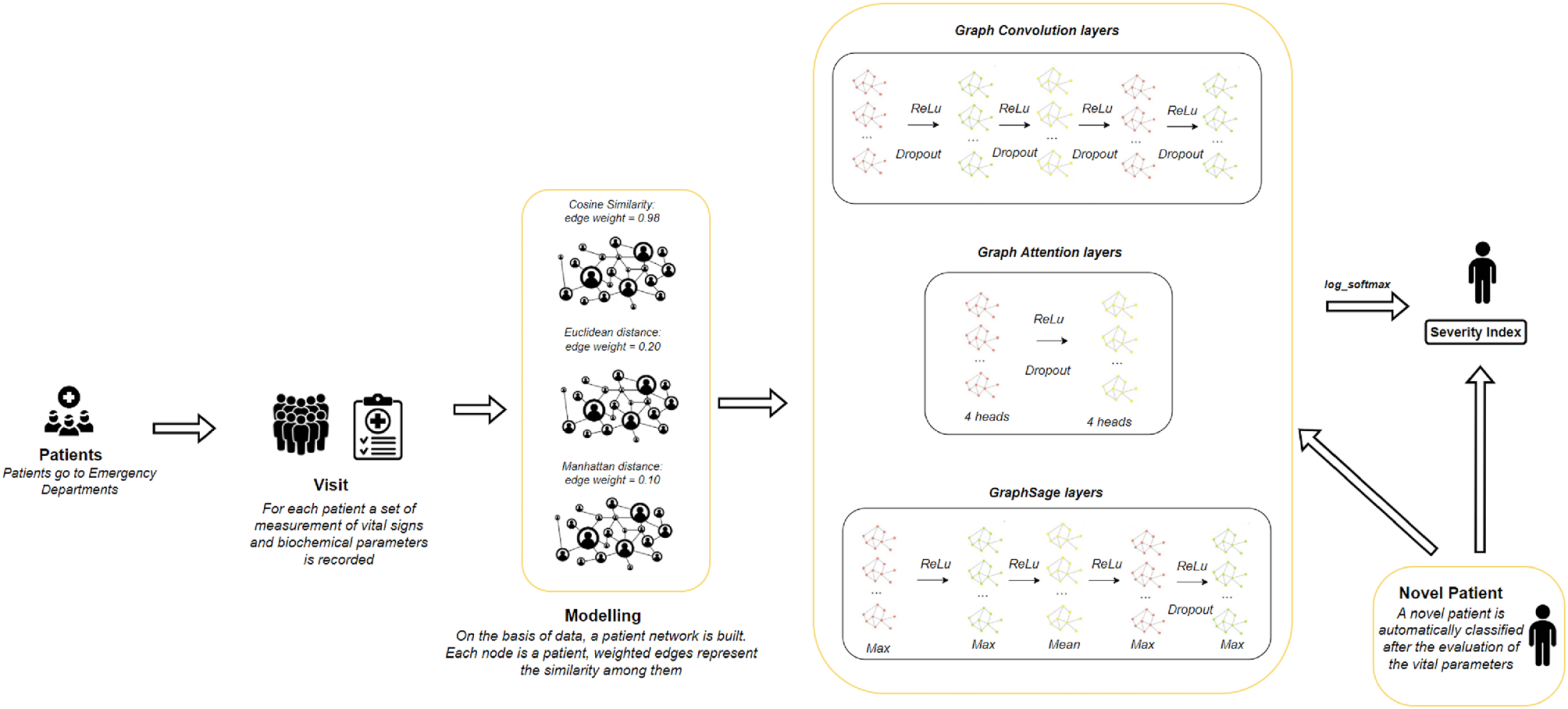
The figure represents the experiments we performed to test the approach. The input dataset is converted into a network using cosine similarity, Euclidean, Manhattan, and Minkowski Distances. For each measure, we generated a set of networks using different thresholds. Then, each single network is used to learn a classifier, and finally, results are evaluated.

To test the performances of our method, we designed and performed a set of experiments as depicted in Fig. 3. The input dataset is translated into networks using cosine similarity, Minkowski, Manhattan, and Euclidean distance. We built many networks for each measure using different threshold levels. Then, for each network, we perform node embedding, and we train a a classifier using GCN, GAT and GraphSage. Finally, we evaluated the performance of each classifier.

### Dataset

We tested our methods on a publicly available dataset on the Kaggle platform (https://www.kaggle.com/datasets/hossamahmedaly/patient-priority-classification). The dataset contains 6962 instances (rows) of patient admission and 16 features for each instance. Each row describes the parameters of a patient such as symptoms and some biochemical parameters used to determine the severity level as summarised in Table 3. Each row also contains the assigned triage level as follows with decrescent level of risk:

- *Red*: the patient needs immediate attention;
- *red*: the patient needs intervention in a short time;
- *Yellow*: urgent condition needing interventions that can be deferred;
- *Green*: condition with minor urgency because there are no alterations of vital functions and no critical symptoms.

**Table 3:** Features of the patients.

**Parameter**	**Description**
Age	age of the patient.
Gender	patient’s sex.
Chest pain type	the type of chest pain.
Blood pressure	blood pressure value.
Cholesterol	cholesterol level.
Max heart rate	maximum heart rate value.
Exercise angina	presence of angina.
Plasma glucose	glucose level in blood plasma.
Skin thickness	any thickening of the skin.
Insulin	insulin level.
BMI	body mass index
Diabetes Pedigree	genetic predisposition to diabetes.
Hypertension	elevated blood pressure.
Heart disease	presence of heart disease.
Residence type	type of residence place.
Smoking status	defines whether or not the patient is a smoker.
Residence type:	Urban,Rural
Smoking status:	never smoked,smoke, previously smoked,Unknown.

**Figure 4:**
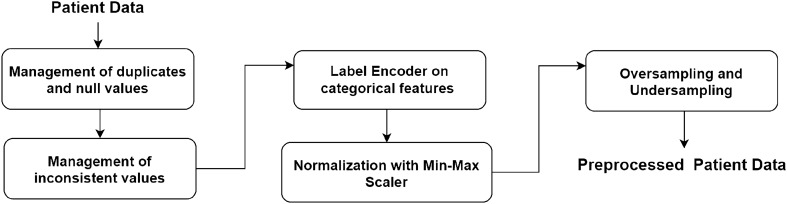
Figure reports the preprocessing workflow.

Data are pre-processed as follows (as reported in Fig. 4):

1. *Duplicates and null values*: this phase involved removing 5.9% of the data. Null values amounted to 411 rows, of which 410 had missing triage codes (with a NaN value) and 1 was missing gender information. Consequently, before implementing other pre-processing phases, 6551 rows remained, accounting for 94.1% of the initial dataset.
2. *Inconsistent (or incomplete) records*: missing values are replaced with the mode of the values of the whole corresponding column.
3. *Label Encoder for categorical features*: Dataset contains three categorical features: *Residence type*, *Smoking status* and the third is the target feature.
4. *Oversampling and Undersampling*: Since classes are unbalanced, we use SMOTE (Synthetic Minority Oversampling Technique)^59^ to perform the sampling.
5. *Normalization with Min-Max Scaler*: Finally data are normalized using a Min-Max approach, so a maximum value of each column is equal to 1 and a minimum value equal to 0.

### Networks generated using cosine similarity

We first generated patient networks using cosine similarity to define edges. We used different thresholds (0.98, 0.95, 0.94, 0.92, 0.90). In this case, an edge connects two nodes when the cosine similarity exceeds the threshold. We stopped at 0.90 since we reached a completely connected graph. Table 4 summarises the characteristics of the networks generated by using cosine similarity at different level of threshold.

**Table 4:** Characteristics of the networks generated using different threshold levels and cosine similarity levels as measure.

Threshold	Isolated Nodes	Edges
**0.98**	**761**	**1.578.490**
**0.95**	**22**	**8.103.196**
0.94	7	10.810.687
0.92	2	16.505.521
0.90	1	22.148.695

Bold highlights best performances.

As can be noticed, decreasing the value of the thresholds reduces the isolated nodes and increases the number of edges. In this way, it is possible to connect more patients due to the possibility of creating an edge between two nodes with some more dissimilarities.

### Networks generated using Euclidean distance

We first generated patient networks using Euclidean Distance to define edges. We used different thresholds (0.20, 0.23, 0.25,0.28,0.31, 0.38). In this case, an edge connects two nodes when the Euclidean distance is lower than the threshold. We stopped at 0.38 since we reached a completely connected graph.

Table 5 summarises the characteristics of the networks generated by using Euclidean Distance at different levels of threshold.

**Table 5:** Characteristics of the networks generated using different threshold levels and Euclidean Distance as similarity measure.

Threshold	Isolated Nodes	Edges
**0.20**	**125**	**3.348.231**
**0.23**	**33**	**5.403.317**
0.25	14	7.120.567
0.28	5	10.279.280
0.31	0	14.086.639
0.38	0	25.226.436

Bold highlights best performances.

The considerations are similar to the case concerning cosine similarity, with the difference that the number of isolated nodes is lower than the first obtained previously. It may be associated with considering distance allows for more connections between a larger number of nodes.

### Networks generated using Manhattan distance

Table 6 summarises the characteristics of the networks generated by using Manhattan Distance at different level of threshold.

**Table 6:** Characteristics of the networks generated using different threshold levels and Manhattan Distance as similarity measure.

Threshold	Isolated Nodes	Edges
**0.10**	**947**	**1.186.583**
**0.13**	**175**	**2.838.619**
0.22	0	14.688.023
0.31	0	35.306.331
0.33	0	41.217.110

Bold highlights best performances.

In this scenario, the number of edges is initially lower than in previous cases, as is the number of isolated nodes. On the contrary, after the first two, much less stringent thresholds were chosen, causing a substantial increase in the number of edges and a reduction to zero in the number of isolated nodes.

### Networks generated using Minkowsky distance

In this subsequent phase, it is interesting to generate graphs from tabular data, by exploring the Minkowski distance. It serves as a generalization of both Euclidean and Manhattan distances. Therefore, it is appropriate to vary the value of p, which characterizes the modification of this metric. Table 7 summarises the network characteristics generated by using Minkowsky Distance (p = 10) at different level of threshold.

**Table 7:** Characteristics of the network generated by using different levels of thresholds and Minkowsky Distance (p = 10) as similarity measure.

Threshold	Isolated Nodes	Edges
**0.20**	**892**	**988.835**
**0.25**	**225**	**2.146.676**
0.30	83	3.942.436
0.35	37	6.403.802
0.40	13	9.481.995

Bold highlights best performances.

Table 8 summarises the network characteristics generated by using Minkowsky Distance (p = 4) at different level of threshold.

**Table 8:** Characteristics of the network generated by using different levels of thresholds and Minkowsky Distance (p = 4) as similarity measure. Significant values are in bold.

Threshold	Isolated Nodes	Edges
**0.20**	**394**	**1.630.369**
**0.25**	**92**	**3.527.312**

It may be observed that choosing p = 10 results in a notable reduction in network connections, differently from choosing p = 4 in which the values are closer to other measures.

### Graph convolutional networks

We used GCN to analyze graphs created based on Cosine similarity and Manhattan on Euclidean distance. We employed two architectures of GCN, the first one for cosine and Manhattan and the last for the networks generated using Euclidean distance.

The first architecture is composed by five GCNConv layers:

- layer 1 of dimensions (16,64), where the first one represents the input feature dimension for each node in the graph: each node is described by a vector of 16 features.
- layers 2,3,4 of dimensions (64,64) representing hidden layers’ dimension during GCN convolutions, followed by dropout function with a fraction of characteristics to be reset equal to 20%, during training and ReLu function for each layer.
- layer 5 of dimensions (64,4) with an output dimension appropriate to the number of classes to be identified as targets.

Similarly, the second architecture is composed, excluding the fifth layer and using 32 as the hidden dimension during GCN convolutions. The input size is set to 16 (in agreement with the number of features), while the output equals 4 (in agreement with the number of feature target classes). Furthermore, the same number of Dropout layers are inserted with the same fraction of characteristics to be reset.

### Graph attention networks

The analysis module based on Graph Attention Network has been implemented as follows with two GATv2Conv layers:

- first layer with input dimension equal to 16 as the number of features, output dimension equal to 8 and 4 attention heads to implement the mechanism of attention.
- second layer with input dimension equal to the output dimension of the previous layer (hidden dimension * number of attention heads) to obtain the output of dimension 4 as the size of the target class.

The two Dropout layers have been introduced at a rate of 20%

### GraphSage

Firstly, the available data is further subjected to a phase which consists of the creation of batches to be able to apply the training on the mini-batches that are better manageable for training the GNN. The *torch geometric.loader* module provides the *Neighborloader* function with the possibility to choose both the size of each batch and the number of neighbours to consider at each iteration. Five sub-graphs are obtained for each considered graph choosing a dimension equal to 3000 for the batch size and some neighbours equal to 10 to be considered at each iteration for each node for 5 iterations. For all the graphs created by the similarity and distance measures, the same model is implemented using five SAGEConv layers. SAGEConv is better than simple GraphSage allowing to capture further details regarding the graph structure, thanks to its aggregate representation based on the degree of the nodes in the neighborhood. In the current implementation, each layer has an input dimension equal to the output dimension of the previous layer, with a sequence of 64, 32, 16, 8. Naturally, the input dimension of the first layer always reflects the number of features, and the output dimension of the last layer corresponds to the number of possible values for the target. Each SAGEConv layer uses the max pooling aggregation function, except for the third layer with the mean aggregation function. Similar to the architectures described earlier, each layer applies a ReLU activation function after the aggregation. Also, a dropout of 20% is applied, but only after the fourth layer.

### Classification performances

**Figure 5:**
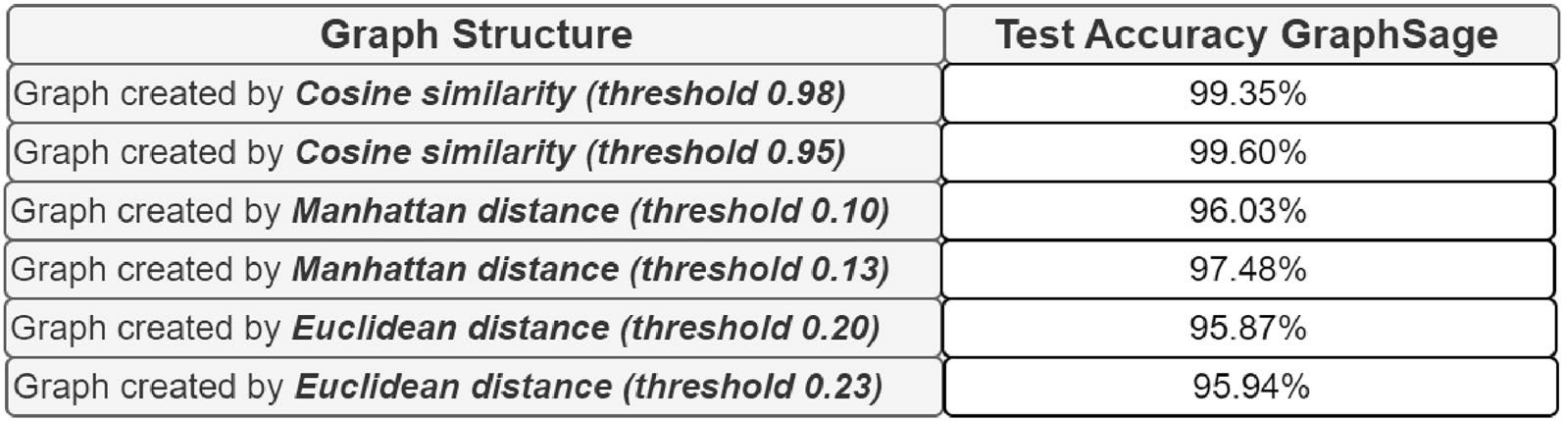
Comparison of test accuracy considering an additional threshold for each metric.

**Figure 6:**
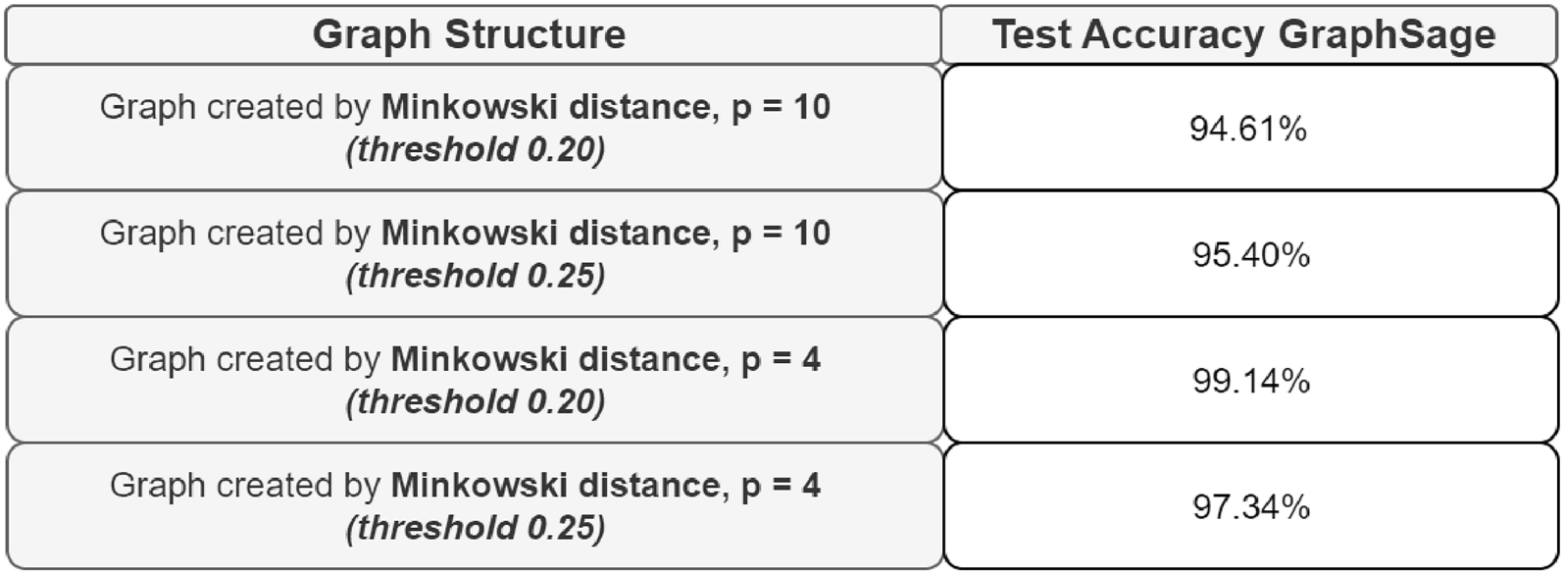
Comparison of test accuracy considering an additional metric.

We measured the classification performances for each network previously built. This helped us to study how some variations in the parameters of the system affect the conclusions of a model. Indeed, the obtained results are visualized in Figs. 5 and 6.

Additionally, it can be observed that for all the considered metrics, opting for a less strict threshold enhances the model’s performance. The only exception is the Minkowski distance with p=4, for which the performance slightly decreases. For this reason, further experiments were conducted with additional arbitrary thresholds, focusing only on the Minkowski distance with p = 10 and excluding p = 4.

**Figure 7:**
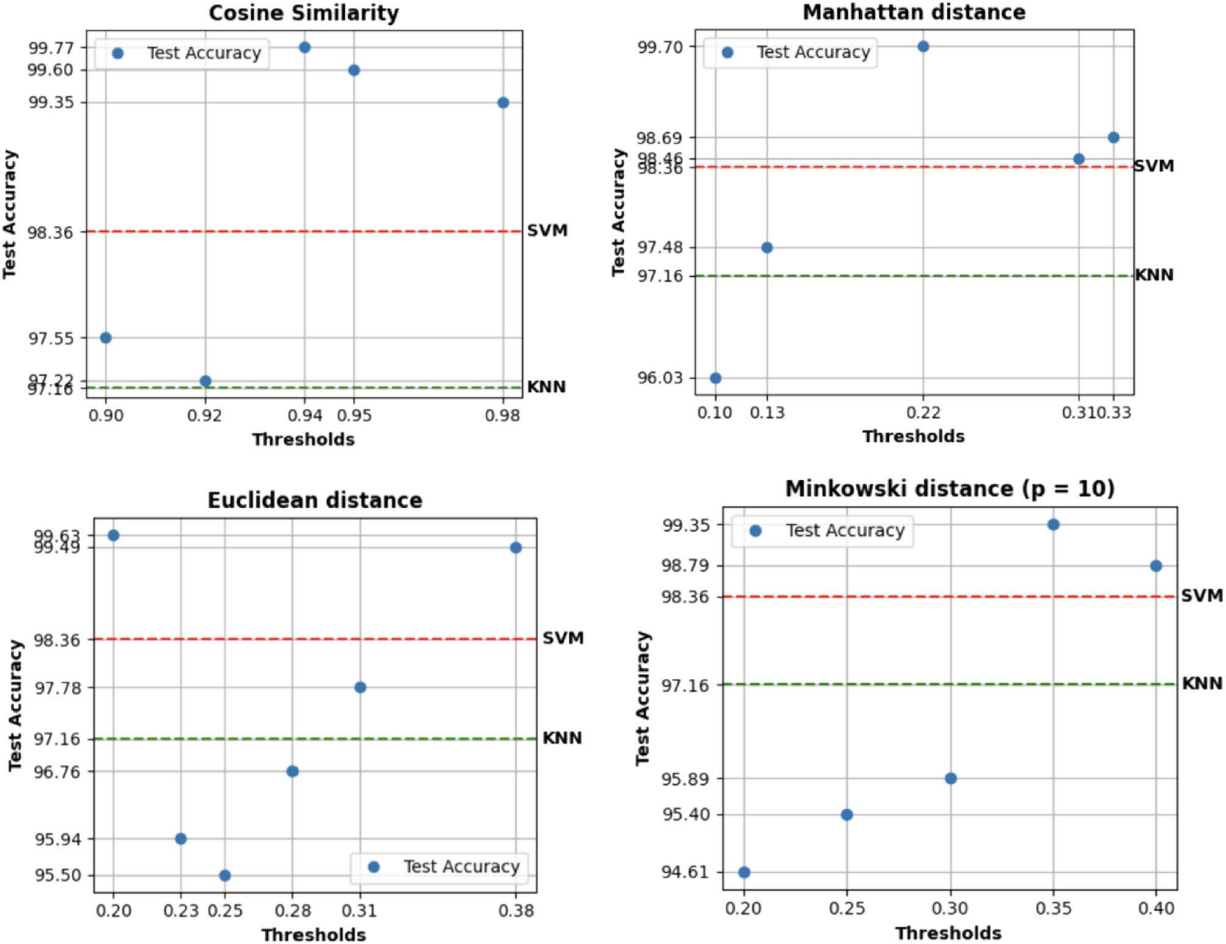
Comparison of test accuracy in relation to the threshold value used for edge creation in each network for each considered metric.

Based on the preliminary results obtained, increasing the number of threshold points used to create the graph structures shown in the previous tables for each metric seems appropriate. The aim will be to display the performance growth or the performance loss. All the values of the arbitrarily considered thresholds are reported in the previous tables, while in the following section, the resulting performances are presented as illustrated in Fig. 7.

For each experiment considering a different metric, some thresholds negatively influenced the models’ performances. These models underperform compared to the others. In addition, the results are worse also than the baseline. On the contrary:

- For the cosine similarity, choosing a threshold lower by a few points is positive for the performances of the model, maybe because it helps to find more similarities useful for the classification. On the other hand, if the threshold is lower than 0.94, it isn’t good.
- Differently from the previous case, an intermediate value of Manhattan distance could be the better choice, outperforming also the baseline.
- Considering the Euclidean distance, higher thresholds deteriorate the results in some cases, as it can be seen more clearly for thresholds like 0.23 and 0.25.
- Ultimately, for the Minkowski distance, to outperform the baseline, the model requires thresholds to be increased in relation to the initial values.

### Node classification

For all the previous methods, we used the following parameters to build the classifier:

- the CrossEntropyLoss computed between the model predictions and the training labels to measure how well the model is learning.
- the Adam optimizer that is employed to update the model weights with a weight decay = 5e-4 and a learning rate of 0.01. This last one is used in all cases except for the model based on GAT layers, which has been set to 0.005.

### Comparison with respect classification on tabular data

It could be useful to compare the results obtained with networks related to classification on tabular data. Therefore, two types of algorithms were trained: Support Vector Machine and K-Nearest Neighbors on the same patient data used for classification on graphs.

**Figure 8:**
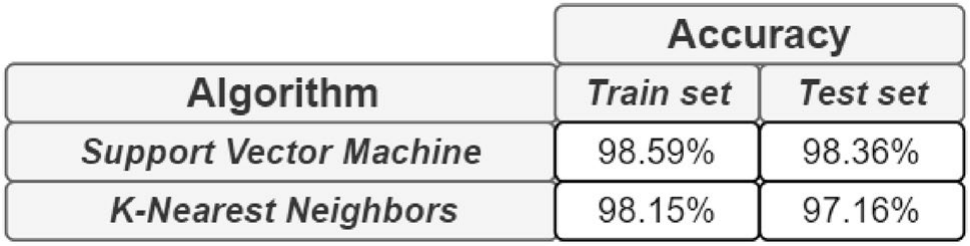
Accuracy on tabular data.

Similarly, the same pre-processing phase was carried out.

The tabular data were splitted in train, test and evaluation set with a proportion of 30% for test set. In addition, a percentage of 30% of the test set was used for evaluation. The results obtained (shown in Fig. 8) were higher than GCN and GAT applied on graph structures but are lesser than GraphSage results. Supporting the evidence, the test performances of the classification on tabular data are also associated to the Sensitivity Analysis results in the previous figures.

### Ablation study

To better test the performances of our approach, we performed an ablatio study^60,61^ considering GraphSage, as it emerged as the best-performing model in the initial analysis phase and using the graph, which resulted in best performances, i.e. cosine similarity with a threshold of 0.95.

**Figure 9:**
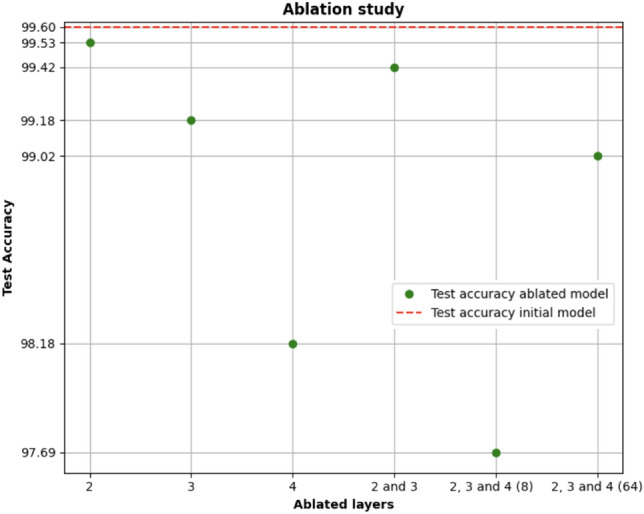
Ablation study results.

In this case, the study was conducted by removing one layer at a time, the second, third, and fourth layers, and finally, all three layers while varying the number of neurons and considering initially eight neurons and then 64 neurons. In Fig. 9, the results obtained are reported, on which it is possible to make the following considerations:

- The removal of the fourth layer slightly reduces performance compared to removing the other layers.
- the removal of the third layer reduces performance slightly more than removing the second layer.
- the removal of the second, third, and fourth layers, considering 8 neurons, leads to the minimum accuracy value achieved in testing.
- The removal of the second, third, and fourth layers, considering 64 neurons, reduces the accuracy value achieved in testing, but it remains higher than that obtained under the same conditions with 8 neurons and higher than that obtained by removing only the second or third layer.

Moreover, it could be argued that the fourth layer may be more important than others in aiding node classification.

### Case study on MIMIC-IV-ED dataset

We also tested our methodology on the demo version of the MIMIC-IV-ED dataset^62,63^ by using the GraphSage configuration since it reported best results in the previous dataset. The dataset we used stores patient data related to triage such as diagnosis, biochemical paratemres and visual inspection.

We selected fourteen features related to triage (’temperature’, ’heartrate’, ’resprate’, ’o2sat’, ’sbp’, ’dbp’, ’pain’, ’gender’, ’race’, ’arrival transport’, ’disposition’, ’name’, ’etcdescription’, ’times minutes’) and reported in the Table 9, to predict the target variable (’acuity’) which has four possible levels (where 1 indicates the highest severity and 4 indicates the lowest severity).

**Table 9:** MIMIC dataset selected features of the patients.

**Parameter**	**Description**
Temperature	patient’s body temperature.
HeartRate	patient’s heart rate
Resprate	respiratory rate.
O2sat	oxygen saturation.
sbp	systolic blood pressure.
dbp	diastolic blood pressure.
Pain	patient reported pain level.
Gender	patient’s sex.
Race	patient’s racial background.
Arrival transport	mechanism of patient admission.
Disposition	patient discharge location.
Name	text description of the medicine.
Etcdescription	the textual description of the ontology group.
Times minutes	the difference between the in-time and out-time^4^.

the difference between the time at which the patient was discharged and the time at which the patient was admitted to the ED.

Preprocessing steps aimed at dropping possible duplicates, imputation of missing values, encoding categorical features, normalisation, and a combined technique of oversampling and undersampling.

**Figure 10:**
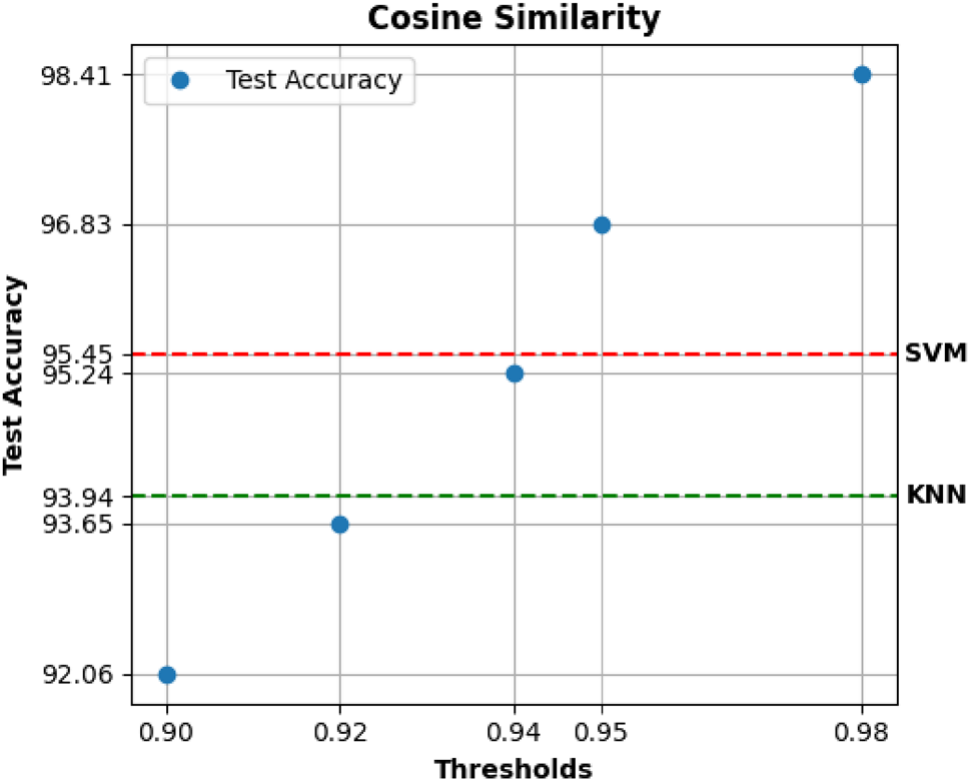
GraphSage’s test accuracy on the MIMIC dataset varies based on the thresholds used for edge creation using Cosine similarity as measure. In addition, it is compared with a standard pipeline’s accuracy applied for the same task.

We initially build the Patient Networks by using Cosine Similarity as measure using five different thresholds (0.90, 0.92, 0.94, 0.95, 0.98). Then we used GraphSage to embed the network and build a classifier. Model’s performances are visible in Fig. 10 showing GraphSage ability in testing. We compared the obtained results with a baseline built as in the previous section. As shown, GraphSage demonstrates good performance, superior to that of the pipeline tested considering higher thresholds for creating networks.

## Discussion

The use of AI-based triage system in EDs could have significant implications for emergency medical care. Automating the triage process could reduce wait times and ensure that patients receive care proportional to the urgency of their condition. This could improve patient outcomes and operational efficiency within hospitals. This study demonstrates the potential of graph neural networks (GNNs) to improve patient triage in emergency departments (EDs) through the application of artificial intelligence (AI) in healthcare. The AI-based module uses historical data and real-time patient information to streamline the triage process and enhance the accuracy of emergency code assignments. In this section, we will provide a detailed analysis of the study’s implications, its inherent strengths and limitations, and propose directions for future research. As discussed in the previous sections, modelling patients in similarity networks and using Graph Neural Networks for embedding and classification showed a high accuracy in predicting triage code. Moreover, using GNNs allows for a more nuanced understanding of patient data, taking into account the complex interrelations between various medical parameters. Our approach leverages vast amounts of data collected in EDs to inform triage decisions, a clear strength of the study. Analysing complex datasets allows for the assessment of each patient’s condition, potentially uncovering correlations that would be missed by human operators.

While it’s true that manual triage times may not be so long, our proposed method addresses critical issues of undertriage and overtriage. This not only supports medical staff in their work esnuring necessary care for patients but saving time and resources. In fact, this data-driven method of triage reduces subjectivity and variability, leading to more consistent and reliable patient care, potentially saving lives through earlier intervention. Despite multiple advantages widely discussed, this approach introduces some limitations regarding mostly the quality and the consistency of the input data. Data incompleteness, inaccuracies, or biases can significantly impact the algorithm’s performance. Additionally, the static nature of historical data may not always capture the dynamic changes in a patient’s condition, underscoring the need for real-time data integration into the AI-based triage system. Even though modelling patients in similarity networks allows to take into account hidden relationships, another limitation is the algorithm’s interpretability. The “black box” nature of AI and machine learning models can make it challenging for medical staff to understand the rationale behind specific triage decisions. This lack of transparency could hinder the acceptance and trust in AI-based triage systems among healthcare professionals.

Although AI systems like the one we propose prove to be accurate and efficient in many areas of medicine and in particular in the specific case of triage, there are certain aspects of the triage process (or other complex medical situations) that may require human judgment. However, automated triage, like the proposed method, could be integrated into clinical practice with appropriate caution, aiding in patient prioritization. About that the integration could optimize decision-making thanks to a data-driven approach that allows to consider similar conditions in patients. In addition, quality of care could be improved due to better resource allocation reducing the waste of resources due to overtriage.

Therefore, while it is an algorithm that leads to the assignment of the triage code, a joint prioritization of human experience and training knowledge of the model could speed up the procedures necessary for the patient’s well-being.

We claimed that the proposed method showed high performance in predicting triage code. The comparison between manual and automatic triage may require the availability of data related to wrong triage, which are not easily available. Future research should explore the integration of real-time monitoring data into the AI-based triage system. Incorporating data from wearable devices, sensors, and other IoT devices could provide a more dynamic and accurate picture of a patient’s condition, allowing for timely adjustments in their triage status.

Moreover, enhancing the interpretability and transparency of AI models is crucial for their adoption in clinical settings. Developing explainable AI (XAI) methods that provide insights into the decision-making process of GNNs could build trust among medical professionals and facilitate the integration of AI systems into existing medical workflows.

Additionally, the standardization and interoperability of medical data formats are vital for the widespread adoption of AI based triage systems. Ensuring that these systems can seamlessly integrate with various hospital information systems and electronic health records (EHRs) will be crucial for their effectiveness and scalability.

## Conclusion

In this study, we introduced an innovative approach for managing patient triage in emergency departments by applying artificial intelligence (AI) and network science. Utilizing machine learning algorithms and graph neural networks, our team crafted an AI module to precisely categorise patients into triage levels. In our tests, this system exhibited superior performance over conventional triage methods.

The findings from our research underscore the advantages of AI integration in healthcare, especially in the context of patient triage. This technological integration streamlines resource allocation and minimizes errors in triage assessments. Our AI-driven method, which analyzes a patient’s detailed medical history alongside their current vital statistics, enables a more detailed and accurate evaluation of their immediate medical needs. This approach significantly enhances the prioritization process of patients within emergency departments.

Moreover, our innovative strategy of representing patient data as graph nodes and employing graph neural networks for classification marks a notable advancement in medical informatics. This technique boosts the precision of patient triage and paves the way for novel methods of analyzing patient data in emergency healthcare scenarios.

Our study illuminates the transformative potential of merging AI with conventional healthcare methodologies to enhance patient care and emergency departments’ operational efficiency. Adopting AI-based systems in healthcare promises redefining triage processes, ensuring more effective and optimized patient treatment. Looking ahead, the focus could be on refining these AI models and examining their applicability in diverse healthcare environments, which could further substantiate and expand upon the observed benefits of this study.

## Data Availability

All the data used in this article and the code for reproducing the experiments are available at https://github.com/hguzzi/iatriage.git.

## References

[1] Moschetti K, Iglesias K, Baggio S, Velonaki V, Hugli O, Burnand B, Daeppen J-B, Wasserfallen J-B, Bodenmann P (2018). Health care costs of case management for frequent users of the emergency department: Hospital and insurance perspectives. PLoS ONE.

[2] Combi, C., Facelli, J. C., Haddawy, P., Holmes, J. H., Koch, S., Liu, H., Meyer, J., Peleg, M., Pozzi, G. & Stiglic, G. *et al.*, The ihi rochester report 2022 on healthcare informatics research: Resuming after the covid-19. *J. Healthcare Inform. Res.* 1–34 (2023). 10.1007/s41666-023-00126-5 PMC10150351 37359193

[3] Parmeggiani C, Abbate R, Marinelli P, Angelillo IF (2010). Healthcare workers and health care-associated infections: Knowledge, attitudes, and behavior in emergency departments in Italy. BMC Infect. Dis..

[4] Savioli G, Ceresa I F, Gri N, Bavestrello Piccini G, Longhitano Y, Zanza C, Piccioni A, Esposito C, Ricevuti G, Bressan M A (2022). Emergency department overcrowding: Understanding the factors to find corresponding solutions. J. Personal. Med..

[5] Bambi S, Becattini G, Ruggeri M (2021). The new emergency department “tuscan triage system”. validation study. Int. Emerg. Nurs..

[6] Cremonesi P, di Bella E, Montefiori M, Persico L (2015). The robustness and effectiveness of the triage system at times of overcrowding and the extra costs due to inappropriate use of emergency departments. Appl. Health Econ. Health Policy.

[7] Boissin C (2022). Clinical decision-support for acute burn referral and triage at specialized centres-contribution from routine and digital health tools. Glob. Health Action.

[8] Defilippo A, Bertucci G, Zurzolo C, Veltri P, Guzzi PH (2023). On the computational approaches for supporting triage systems. Interdiscip. Med..

[9] Hinson JS, Martinez DA, Cabral S, George K, Whalen M, Hansoti B, Levin S (2019). Triage performance in emergency medicine: a systematic review. Ann. Emerg. Med..

[10] Murray MJ (2003). The Canadian triage and acuity scale: A Canadian perspective on emergency department triage. Emerg. Med..

[11] Bullard MJ, Musgrave E, Warren D, Unger B, Skeldon T, Grierson R, van der Linde E, Swain J (2017). Revisions to the Canadian emergency department triage and acuity scale (ctas) guidelines 2016. Can. J. Emerg. Med..

[12] Putri AP, Widiyanto A, Handayani RT, Darmayanti AT (2020). Australasian triage scale (ats): Literature review. J. Borneo Holistic Health.

[13] Azeredo TRM, Guedes HM, de Almeida RAR, Chianca TCM, Martins JCA (2015). Efficacy of the Manchester triage system: A systematic review. Int. Emerg. Nurs..

[14] Wuerz RC, Travers D, Gilboy N, Eitel DR, Rosenau A, Yazhari R (2001). Implementation and refinement of the emergency severity index. Acad. Emerg. Med..

[15] Kwon H, Kim YJ, Jo YH, Lee JH, Lee JH, Kim J, Hwang JE, Jeong J, Choi YJ (2019). The Korean triage and acuity scale: Associations with admission, disposition, mortality and length of stay in the emergency department. Int. J. Qual. Health Care.

[16] Ng C -J, Yen Z -S, Tsai J C -H, Chen L C, Lin S J, Sang Y Y, Chen J -C, Group T N W (2011). Validation of the Taiwan triage and acuity scale: A new computerised five-level triage system. Emerg. Med. J..

[17] Meyer GD, Meyer TN, Gaunt CB (2018). Validity of the South African triage scale in a rural district hospital. Afr. J. Emerg. Med..

[18] FitzGerald G, Jelinek GA, Scott D, Gerdtz MF (2010). Emergency department triage revisited. Emerg. Med. J..

[19] Eid W, Borie H (2021). Comparing accuracy of manual triage with electronic triage system. Am. J. Nurs. Res..

[20] Chong, H. & Gan, K. Development of automated triage system for emergency medical service. In *2016 International Conference on Advances in Electrical, Electronic and Systems Engineering (ICAEES)* 642–645 (IEEE, 2016).

[21] Cheung D S, Grubenhoff J A (2019). Machine learning in clinical medicine still finding its way. JAMA Netw. Open.

[22] Canino G, Guzzi PH, Tradigo G, Zhang A, Veltri P (2015). On the analysis of diseases and their related geographical data. IEEE J. Biomed. Health Inform..

[23] Olivia, D., Nayak, A. & Balachandra, M. Machine learning based electronic triage for emergency department. In *International Conference on Applications and Techniques in Information Security* 215–221 (Springer, 2018).

[24] Caicedo-Torres, W., García, G. & Pinzón, H. A machine learning model for triage in lean pediatric emergency departments. In *Advances in Artificial Intelligence-IBERAMIA 2016: 15th Ibero-American Conference on AI, San José, Costa Rica, November 23–25, 2016, Proceedings 15* 212–221 (Springer, 2016).

[25] Joseph JW, Leventhal EL, Grossestreuer AV, Wong ML, Joseph LJ, Nathanson LA, Donnino MW, Elhadad N, Sanchez LD (2020). Deep-learning approaches to identify critically ill patients at emergency department triage using limited information. J. Am. College Emerg. Phys. Open.

[26] Levin S, Toerper M, Hamrock E, Hinson JS, Barnes S, Gardner H, Dugas A, Linton B, Kirsch T, Kelen G (2018). Machine-learning-based electronic triage more accurately differentiates patients with respect to clinical outcomes compared with the emergency severity index. Ann. Emerg. Med..

[27] Leung, K.-C., Lin, Y.-T., Hong, D.-Y., Tsai, C.-L., Huang, C.-H. & Fu, L.-C. A novel interpretable deep-learning-based system for triage prediction in the emergency department: A prospective study. In *2021 IEEE International Conference on Systems, Man, and Cybernetics (SMC)* 2979–2985 (IEEE, 2021).

[28] Hong WS, Haimovich AD, Taylor RA (2018). Predicting hospital admission at emergency department triage using machine learning. PLoS ONE.

[29] Bentéjac C, Csörgő A, Martínez-Muñoz G (2021). A comparative analysis of gradient boosting algorithms. Artif. Intell. Rev..

[30] Tong, C., Rocheteau, E., Veličković, P., Lane, N. & Liò, P. Predicting patient outcomes with graph representation learning. In *International Workshop on Health Intelligence* 281–293 (Springer, 2021).

[31] Kipf, T.N. & Welling, M. Semi-supervised classification with graph convolutional networks. arXiv preprint arXiv:1609.02907 (2016).

[32] Pranathi, K.S. & Prathibhamol, C. Node classification through graph embedding techniques. In *2021 4th Biennial International Conference on Nascent Technologies in Engineering (ICNTE)* 1–4 (IEEE, 2021).

[33] Zitnik, M., Li, M.M., Wells, A., Glass, K., Gysi, D.M., Krishnan, A., Murali, T., Radivojac, P., Roy, S., Baudot, A. *et al.* Current and future directions in network biology. arXiv preprint arXiv:2309.08478 (2023). 10.1093/bioadv/vbae099 PMC11321866 39143982

[34] Guzzi PH, Mina M, Guerra C, Cannataro M (2012). Semantic similarity analysis of protein data: Assessment with biological features and issues. Brief. Bioinform..

[35] Swedish Council on Health Technology Assessment. Triage methods and patient flow processes in emergency departments: A systematic review. Swedish Council on Health Technology Assessment (SBU), SBU Yellow Report 197, April 2010, pMID: 28876773. [Online]. Available: https://www.sbu.se/2010-04-20/. 28876773

[36] Aacharya RP, Gastmans C, Denier Y (2011). Emergency department triage: An ethical analysis. BMC Emerg. Med..

[37] Yu JY, Jeong GY, Jeong OS, Chang DK, Cha WC (2020). Machine learning and initial nursing assessment-based triage system for emergency department. Healthc. inform. Res..

[38] Choi SW, Ko T, Hong KJ, Kim KH (2019). Machine learning-based prediction of Korean triage and acuity scale level in emergency department patients. Healthc. Inform. Res..

[39] Cannataro M, Guzzi PH, Veltri P (2010). Impreco: Distributed prediction of protein complexes. Futur. Gener. Comput. Syst..

[40] Hiram Guzzi P, Petrizzelli F, Mazza T (2022). Disease spreading modeling and analysis: A survey. Brief. Bioinform..

[41] Inokuchi R, Iwagami M, Sun Y, Sakamoto A, Tamiya N (2022). Machine learning models predicting undertriage in telephone triage. Ann. Med..

[42] Allen A, Mataraso S, Siefkas A, Burdick H, Braden G, Dellinger RP, McCoy A, Pellegrini E, Hoffman J, Green-Saxena A (2020). A racially unbiased, machine learning approach to prediction of mortality: Algorithm development study. JMIR Public Health Surveill..

[43] Singh VK, Shrivastava U, Bouayad L, Padmanabhan B, Ialynytchev A, Schultz SK (2018). Machine learning for psychiatric patient triaging: An investigation of cascading classifiers. J. Am. Med. Inform. Assoc..

[44] Yan, S., Peng, J., Grain, H. & Yi, M. Technology road mapping of two machine learning methods for triaging emergency department patients in Australia. In *Proceedings of the 2019 the International Conference on Pattern Recognition and Artificial Intelligence* 60–67 (2019).

[45] An, Y., Li, R. & Chen, X. Merge: A multi-graph attentive representation learning framework integrating group information from similar patients. *Computers in Biology and Medicine*, vol. 151, 106245 (2022). [Online]. Available: https://www.sciencedirect.com/science/article/pii/S0010482522009532 10.1016/j.compbiomed.2022.106245 36335809

[46] Liu, Z., Li, X., You, Z., Yang, T., Fan, W., & Yu, P. Medical triage chatbot diagnosis improvement via multi-relational hyperbolic graph neural network. In *Proceedings of the 44th International ACM SIGIR Conference on Research and Development in Information Retrieval*, ser. SIGIR ’21 1965-1969 (Association for Computing Machinery, 2021). [Online]. Available: 10.1145/3404835.3463095.

[47] Valls V, Zayats M, Pascale A (2023). Information flow in graph neural networks: A clinical triage use case. IEEE Int. Conf. Digit. Health (ICDH).

[48] Reychav I, McHaney R, Babbar S, Weragalaarachchi K, Azaizah N, Nevet A (2022). Graph network techniques to model and analyze emergency department patient flow. Mathematics.

[49] Sun J (2022). Design of intelligent question answering system for hospital online triage based on knowledge graph. Highlights Sci. Eng. Technol..

[50] Guzzi PH, Zitnik M (2022). Editorial deep learning and graph embeddings for network biology. IEEE/ACM Trans. Comput. Biol. Bioinf..

[51] Gu S, Jiang M, Guzzi PH, Milenković T (2022). Modeling multi-scale data via a network of networks. Bioinformatics.

[52] Kumar Das J, Tradigo G, Veltri P, Guzzi P H, Roy S (2021). Data science in unveiling covid-19 pathogenesis and diagnosis: evolutionary origin to drug repurposing. Brief. Bioinform..

[53] Betkier I, Oszczypała M, Pobożniak J, Sobieski S, Betkier P (2023). Pocketfindergnn: A manufacturing feature recognition software based on graph neural networks (gnns) using pytorch geometric and networkx. SoftwareX.

[54] Brody, S., Alon, U. & Yahav, E. How attentive are graph attention networks? arXiv preprint arXiv:2105.14491 (2021).

[55] Hamilton W, Ying Z, Leskovec J (2017). Inductive representation learning on large graphs. Adv. Neural Inform. Process. Syst..

[56] Guzzi PH, Cortese F, Mannino GC, Pedace E, Succurro E, Andreozzi F, Veltri P (2023). Analysis of age-dependent gene-expression in human tissues for studying diabetes comorbidities. Sci. Rep..

[57] Zhang X, Kim J, Patzer RE, Pitts SR, Chokshi FH, Schrager JD (2019). Advanced diagnostic imaging utilization during emergency department visits in the united states: A predictive modeling study for emergency department triage. PLoS ONE.

[58] Velickovic P, Cucurull G, Casanova A, Romero A, Lio P, Bengio Y (2017). Graph attention networks. Statistics.

[59] Chawla NV, Bowyer KW, Hall LO, Kegelmeyer WP (2002). Smote: Synthetic minority over-sampling technique. J. Artif. Intell. Res..

[60] Sheikholeslami, S., Meister, M., Wang, T., Payberah, A. H., Vlassov, V. & Dowling, J. Autoablation: Automated parallel ablation studies for deep learning. In *Proceedings of the 1st Workshop on Machine Learning and Systems* 55–61 (2021).

[61] Meyes, R., Lu, M., de Puiseau, C. W. & Meisen, T. Ablation studies in artificial neural networks. arXiv preprint arXiv:1901.08644 (2019).

[62] Goldberger A, Amaral L, Glass L, Hausdorff J, Ivanov P, Mark R, Mietus J, Moody G, Peng C, Stanley H (2020). Physiobank, physiotoolkit, and physionet: Components of a new research resource for complex physiologic signals. Circulation.

[63] Johnson, A., Bulgarelli, L., Pollard, T., Celi, L., Mark, R. & Horng, S. Mimic-iv-ed (version 2.2). *PhysioNet*. Available at: 10.13026/5ntk-km72 (2023).

